# Supplementary figures and images for: The benefits of insect-swarm hunting to echolocating bats, and its influence on the evolution of bat echolocation signals
Source: PLoS Comput Biol. 2019 Dec 12;15(12):e1006873. doi: 10.1371/journal.pcbi.1006873 (PMC6907744; doi:10.1371/journal.pcbi.1006873)

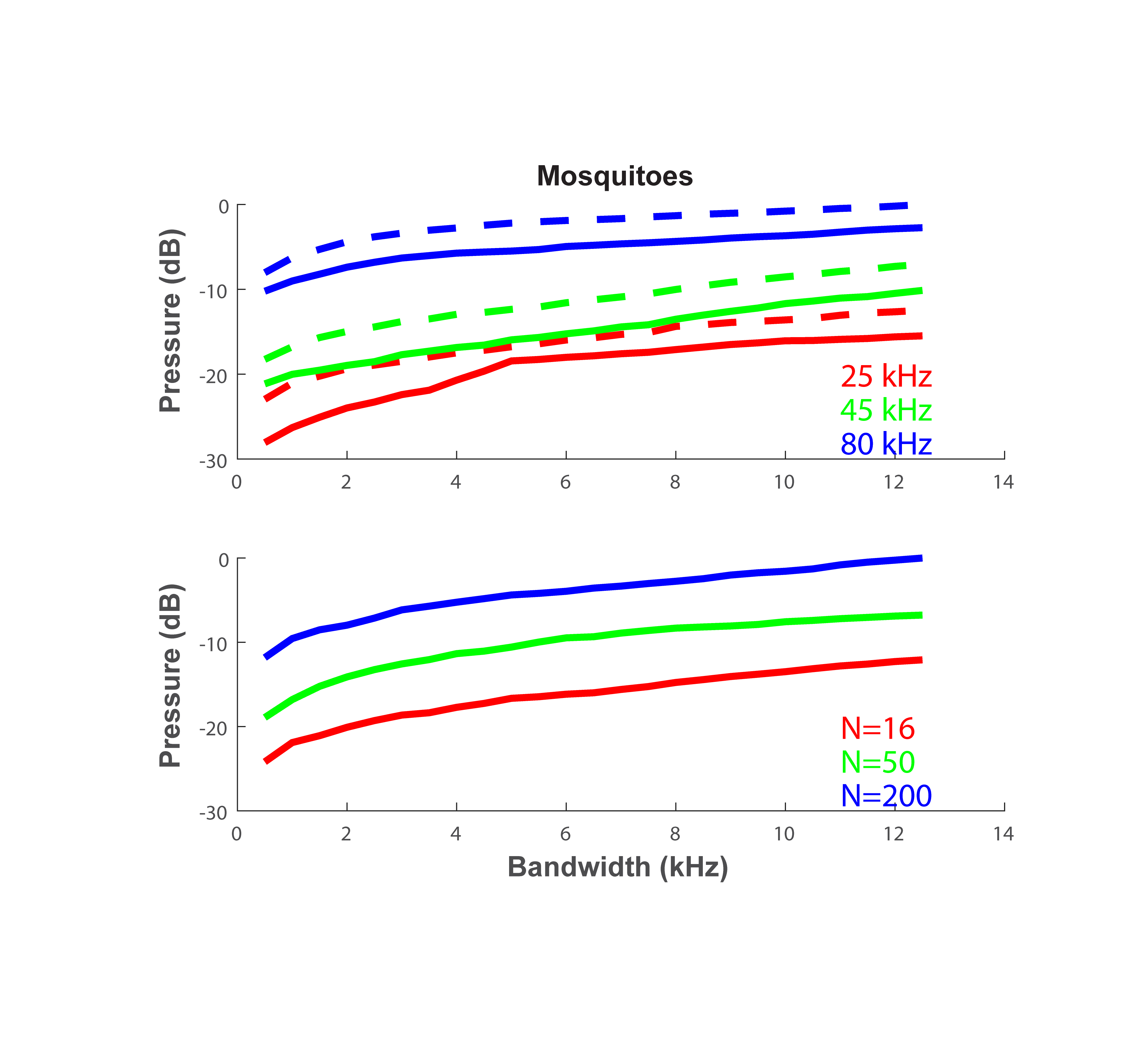

Supplement: S1 Fig — Upper graph: Solid line R = 15mm, dashed line R = 200mm, colors denote different frequencies. Lower graph: R = 35mm, but with different swarm sizes. Results from this simulation reveal similar principles as in Fig 3 in the paper. (TIF) [file pcbi.1006873.s001.tif]

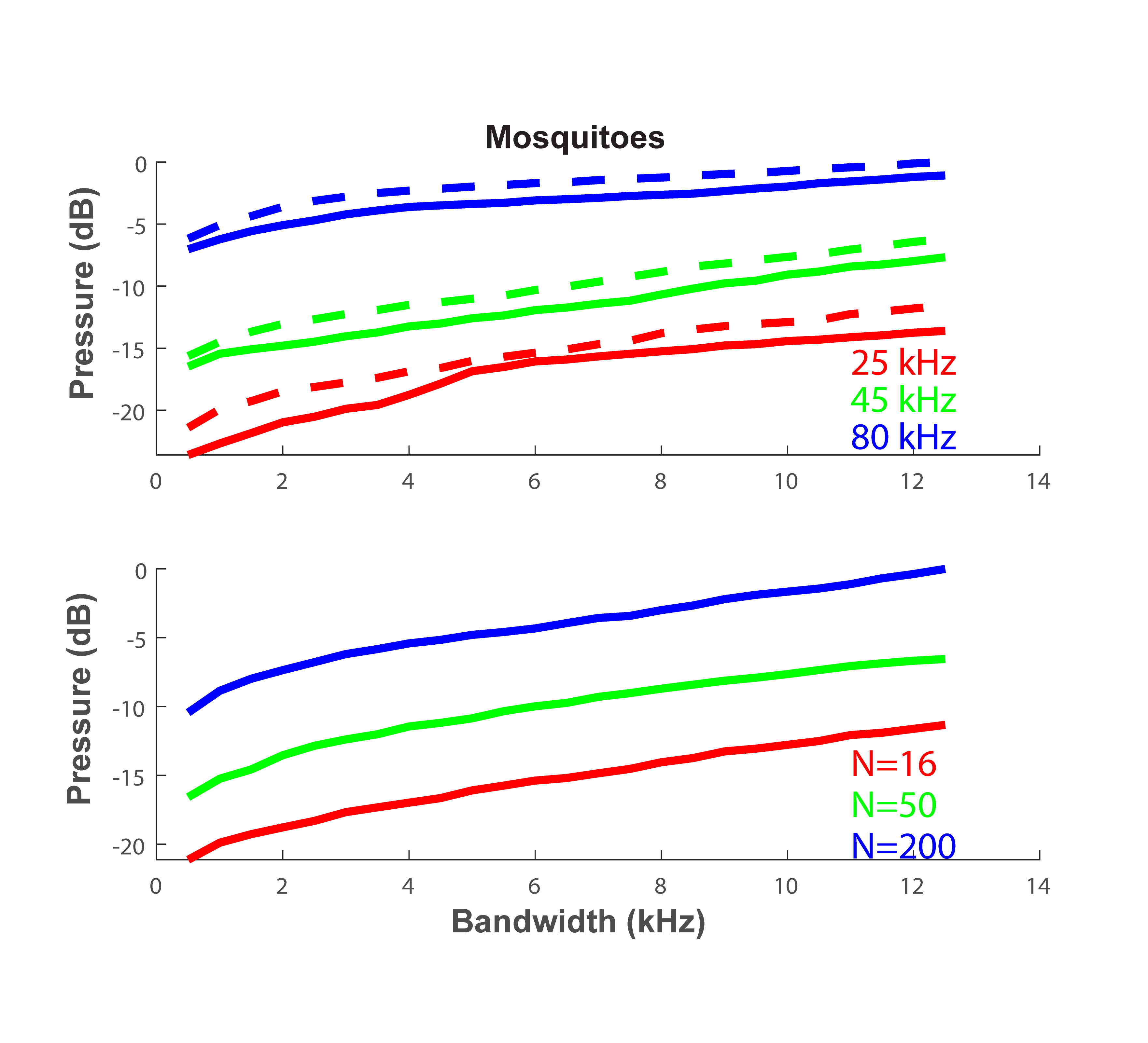

Supplement: S2 Fig — Upper graph: Solid line R = 15mm, dashed line R = 200mm, colors denote different frequencies. Lower graph: R = 35mm, but with different swarm sizes. Results from this simulation reveal similar principles as in Fig 3 in the paper. (TIF) [file pcbi.1006873.s002.tif]

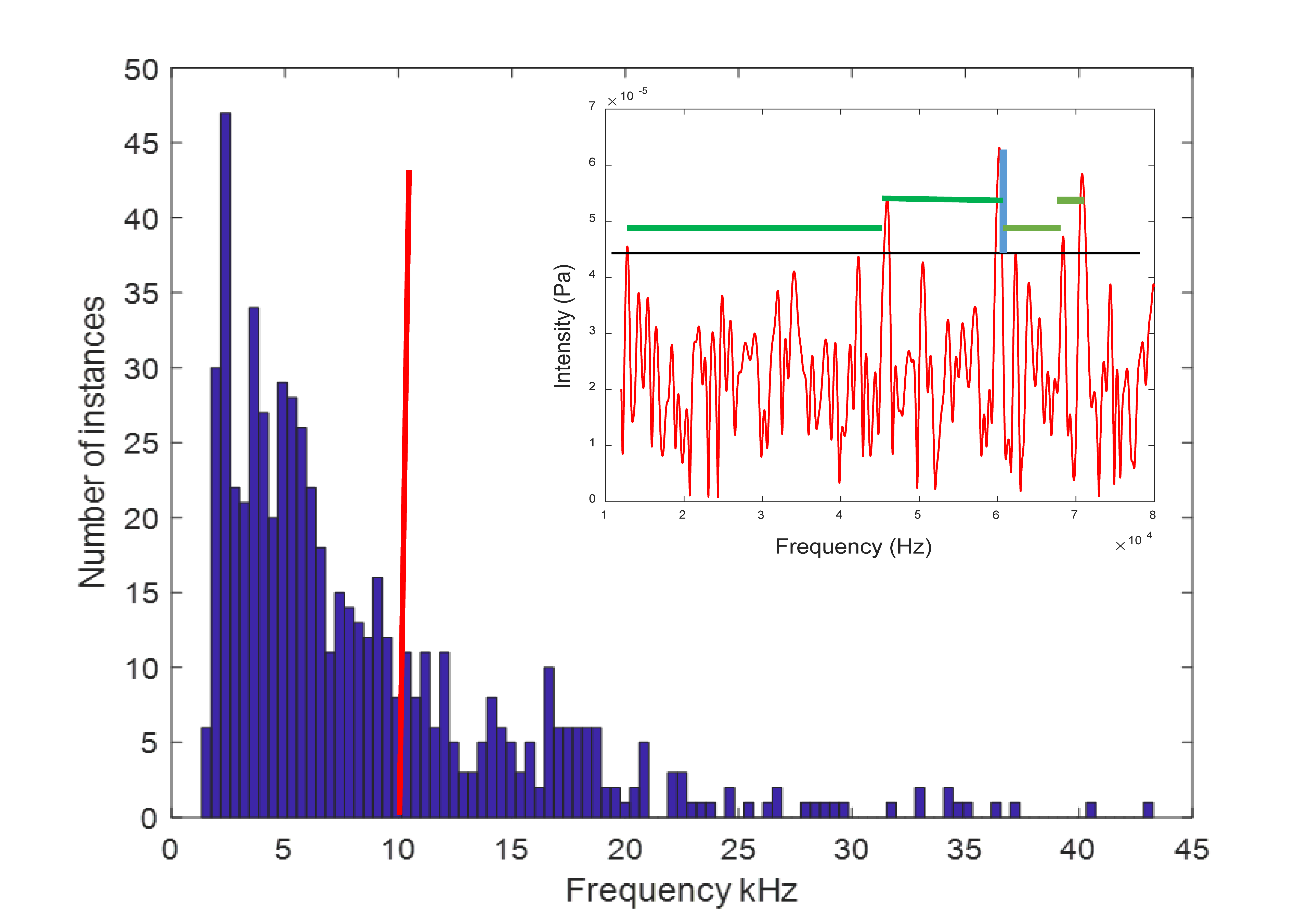

Supplement: S3 Fig — For each spectrum, the bandwidth of all troughs between pairs of peaks was measured. Peaks were defined as peaks that are no more than 20% lower than the maximum of the entire spectrum (black horizontal line inset; green horizontal lines: trough-widths). The bandwidth of a trough can be thought of as the minimal bandwidth allowing to receive a peak pressure for a given swarm realization. Red line shows that a bandwidth of 10 kHz would ensure that the bat receives the maximal echo (i.e., no more than 20% weaker than the absolute possible maximum) for 70% of the echoes (the line parts the histogram to 30:70%). We repeated this analysis for different peak criteria (10, 30, 40%) and the pattern is the same (the bandwidth would obviously change accordingly). We call this point of 70% BWc–the critical bandwidth. (TIF) [file pcbi.1006873.s003.tif]

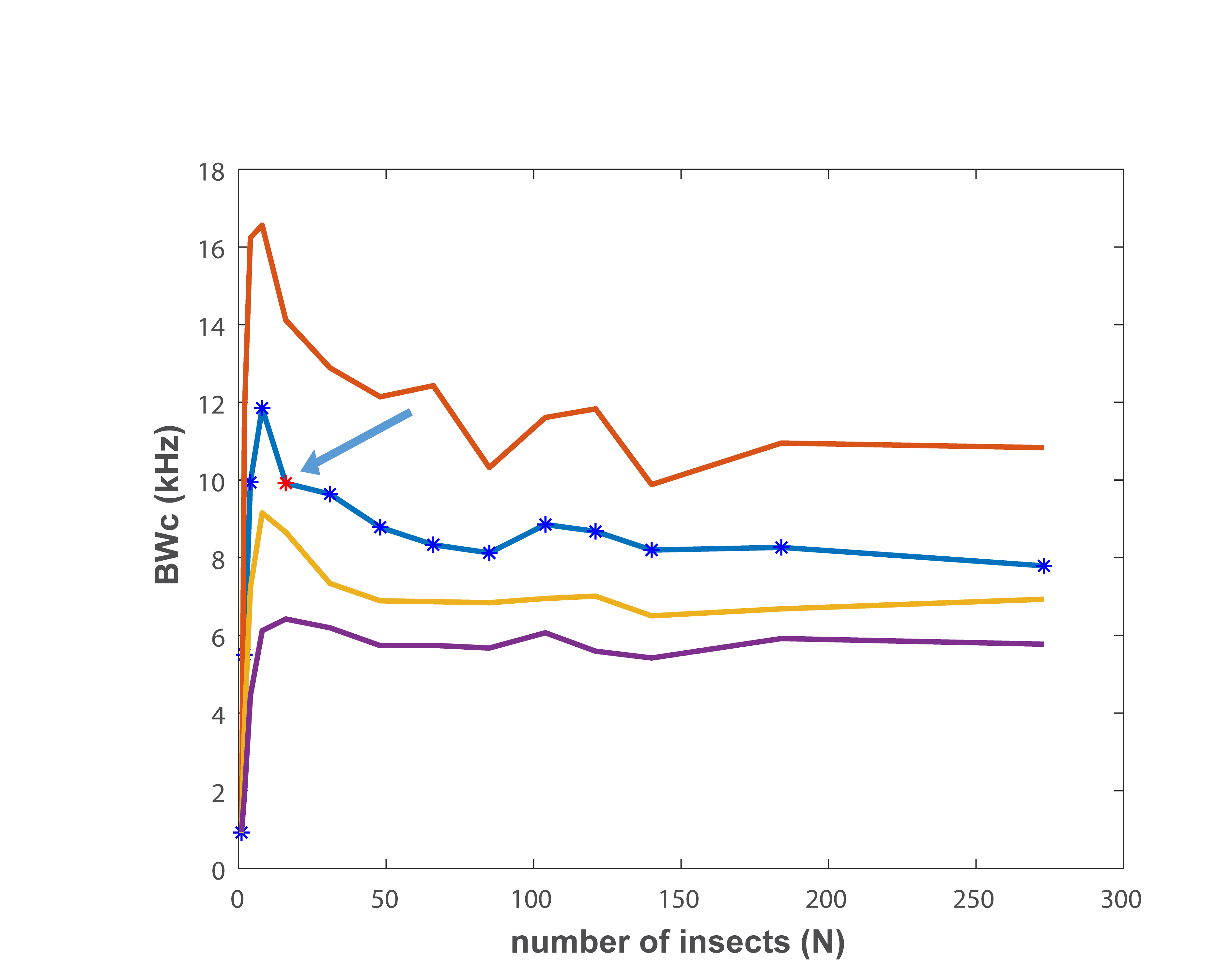

Supplement: S4 Fig — BWc was extracted as in S3 Fig. Red star (on blue line) is the same point as the red line shown in S3 Fig. The same pattern was observed for swarms of different densities (R = 15mm; R = 60mm and 120mm; red, yellow and purple lines respectively). More bandwidth is needed for denser swarms (compare red and purple lines) and less bandwidth is needed to capture spectral peaks of sparser swarms. This can also be learned from Fig 2 in the main text. (TIF) [file pcbi.1006873.s004.tif]

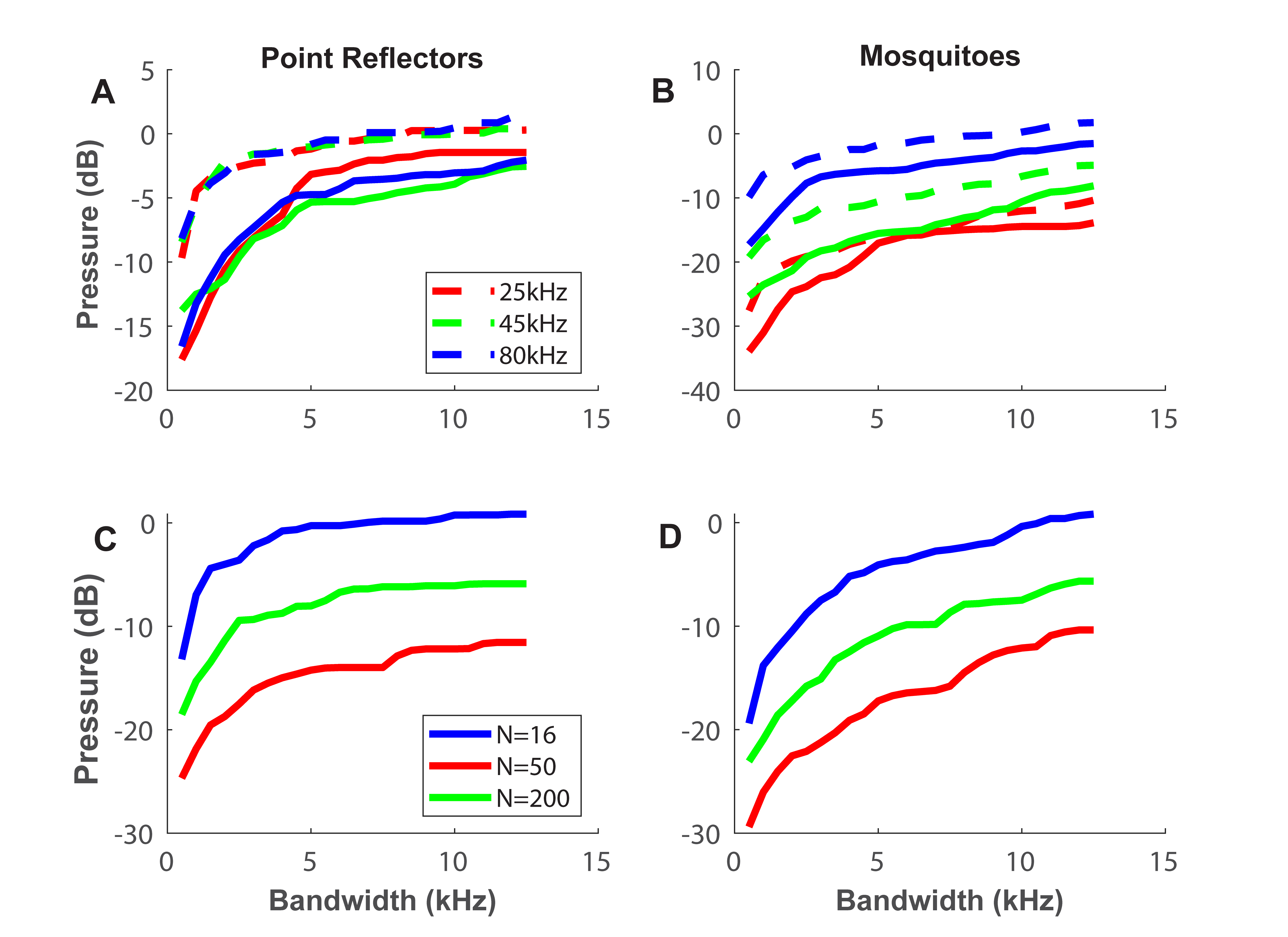

Supplement: S5 Fig — Worst cases: in the minimal bandwidth case (500 Hz), a bat is faced with the worst spectral trough of 100 simulations. The figures show that adding bandwidth improves swarm detection by 15-20dB on average in this worst case scenario. (A) Echo sound pressure as a function of bandwidth for a 100-reflector swarm with three different upper -frequencies (colors) and two different inter-reflector distances (15 and 120mm represented by solid vs. dashed lines respectively). (B) The same as in (A) but for 100-mosquito-like reflectors. (C) The echo sound pressure as a function of bandwidth for a point-reflector swarm with different numbers of reflectors (depicted by different colors). The upper frequency was 25 kHz for these simulations. (D) The same as in (C) but for mosquito-like reflectors. Each point (in all panels A-D) is based on generating 100 stochastic swarm realizations, calculating the loudest peak over the relevant bandwidth (depicted on the x-axis). Note that the lowest bandwidth is not 0 Hz, but 500 Hz, therefore displaying only intended bandwidth and not natural variation (see Discussion). (TIF) [file pcbi.1006873.s005.tif]

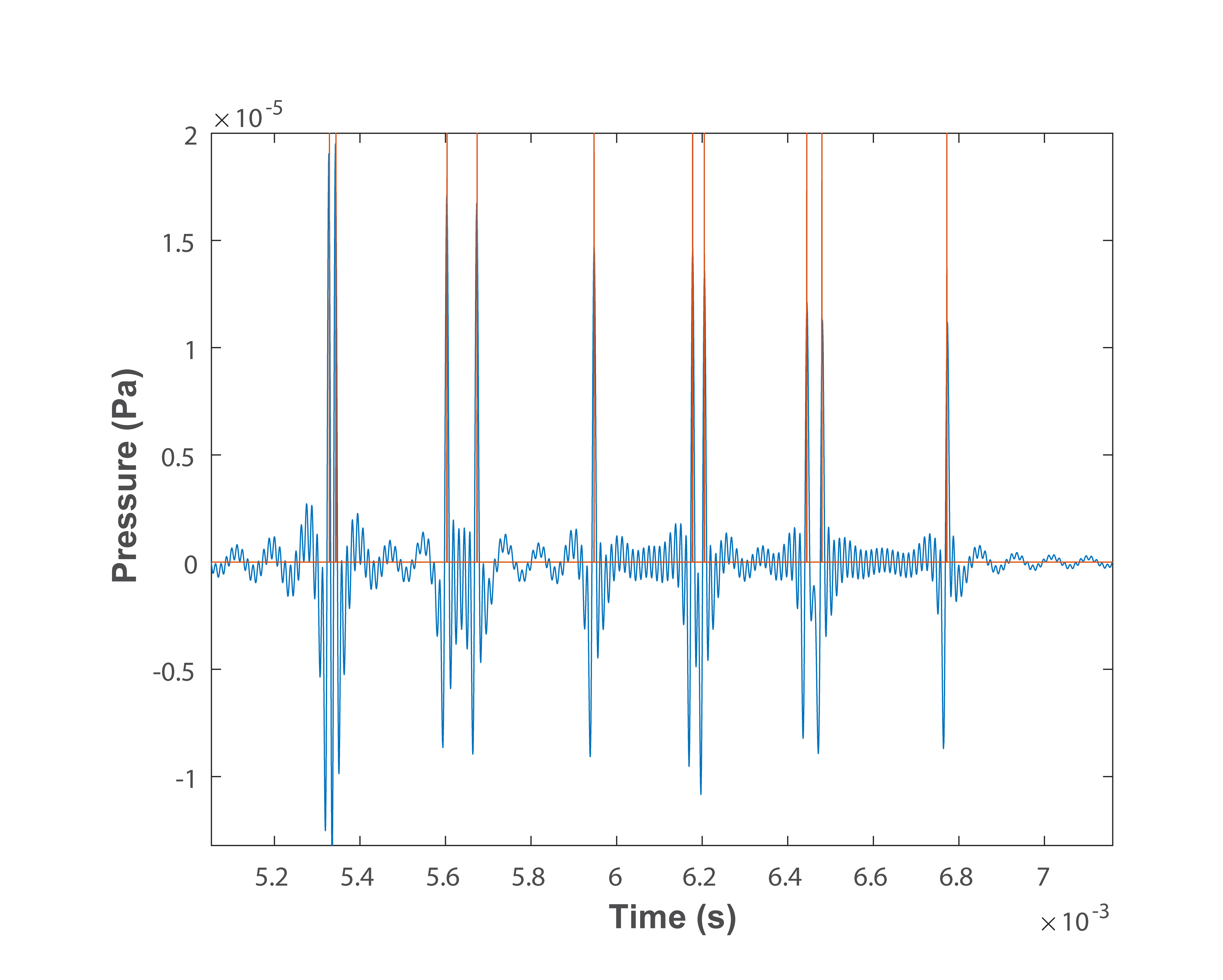

Supplement: S6 Fig — We tested the validity of the analytic model (Eq 1) numerically by generating swarms with 1 to 10 points at known distances Di, calculating the power spectrum of their echoes (using Eq 1), and then estimating the impulse response by means of an inverse Fourier transform. The impulse response preserves the temporal information which can be compared to the distances of the reflectors. The spectra were calculated between 12 to 80 kHz in steps of 100Hz. The figure shows the actual point distances Di for a single swarm with 10-points (red) and the impulse response generated according to Eq 1 (blue). The distances are presented over a time axis to ease the comparison. (TIF) [file pcbi.1006873.s006.tif]

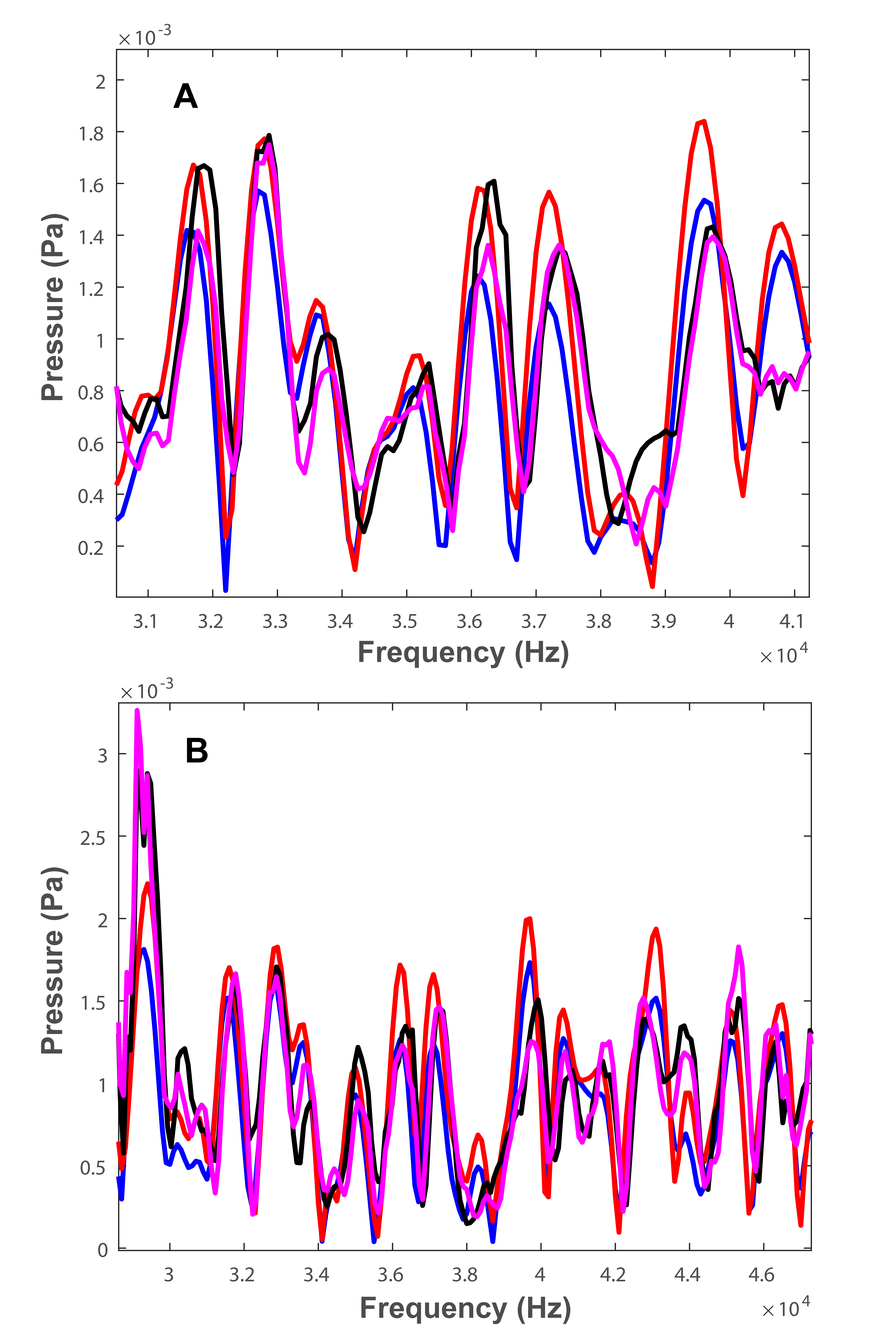

Supplement: S7 Fig — The spectrum of three models is presented: the analytic model (red), the boundary element model (blue) and the actual recording of real beads (in black and in magenta for echoes for which the emission was shifted by 180 degrees). Top: the spectrum of a 7-reflector model is presented. Bottom: the spectrum of an 8-reflector model is presented. For a better comparison, the spectrum between 30–42 kHz is enlarged. Note that the real beads had a 2cm diameter which could explain the slight differences between the actual echoes and the simulated ones. (TIF) [file pcbi.1006873.s007.tif]

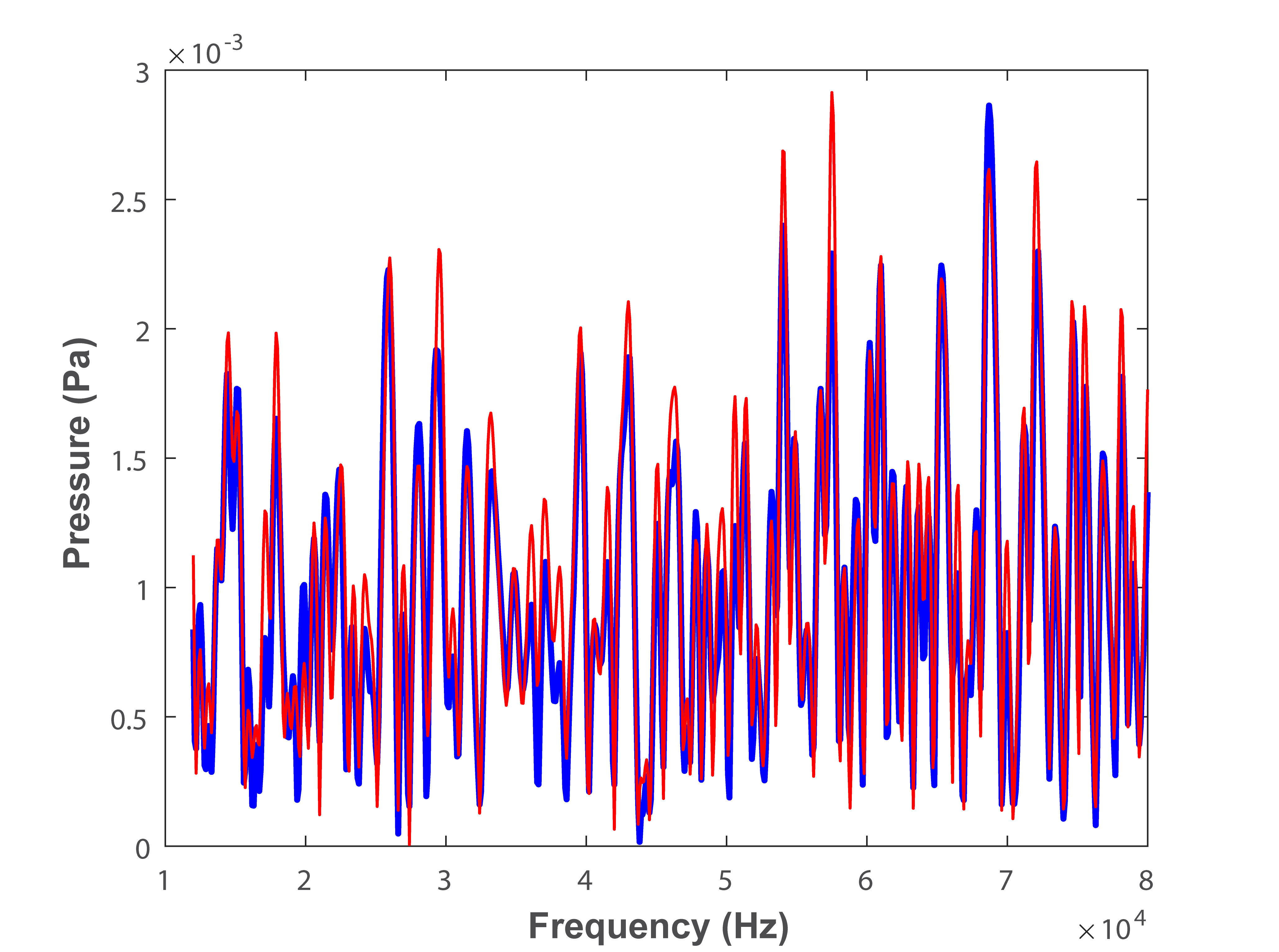

Supplement: S8 Fig — The spectrum of two models is presented for a 10-reflector model: the analytic model (red), the boundary element model (blue). We do not show the data for the physical bead model because it would be difficult to see with another graph overlaid. (TIF) [file pcbi.1006873.s008.tif]

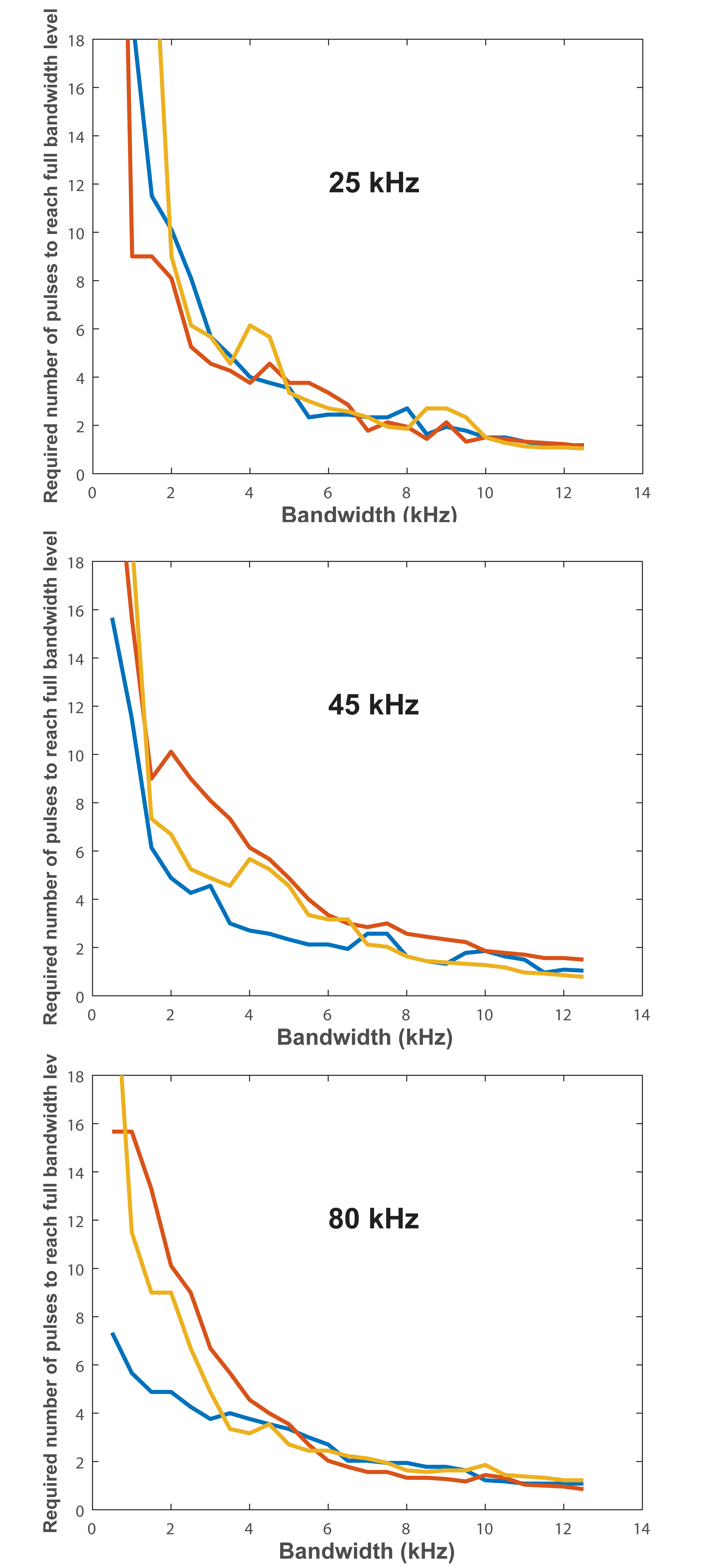

Supplement: S9 Fig — A bat can either increase its bandwidth (Fig 3 this paper) or it can repeat the emission several times to reach the same detection probability of detecting an insect swarm. Blue: swarm size N = 16; Red: N = 50; Orange: N = 200. All graphs representing R = 35mm. Upper frequencies of a signal with 12.5 kHz bandwidth are indicated within each graph. This graph shows how the two entities (bandwidth and repetition) relate to each other. When using bandwidths under 1.5 kHz, the required number of emissions to detect a swarm (detection level defined as the average sound pressure the swarm evokes at full bandwidth) quickly rises to above 10. In practice this means that the bat is losing time and energy (in having to emit more pulses) when not widening bandwidth. The trade-off between bandwidth and number of pulses depicted in this graph was calculated on the basis of 100 swarm simulations for each bandwidth, resulting in a probability distribution of reaching different pressures. For each bandwidth, we calculated the probability of reaching or surpassing the average pressure as received with a 12.5 kHz bandwidth pulse. The inverse of this probability was used to calculate the expected number of pulses (trials) needed to equalize to the performance at this bandwidth. (TIF) [file pcbi.1006873.s009.tif]
